# A Model of Indel Evolution by Finite-State, Continuous-Time Machines

**DOI:** 10.1534/genetics.120.303630

**Published:** 2020-10-05

**Authors:** Ian Holmes

**Affiliations:** Department of Bioengineering, University of California, Berkeley, California 94720

**Keywords:** automata, hidden Markov models, indels, Markov processes, molecular evolution, phylogenetics

## Abstract

We introduce a systematic method of approximating finite-time transition probabilities for continuous-time insertion-deletion models on sequences. The method uses automata theory to describe the action of an infinitesimal evolutionary generator on a probability distribution over alignments, where both the generator and the alignment distribution can be represented by pair hidden Markov models (HMMs). In general, combining HMMs in this way induces a multiplication of their state spaces; to control this, we introduce a coarse-graining operation to keep the state space at a constant size. This leads naturally to ordinary differential equations for the evolution of the transition probabilities of the approximating pair HMM. The TKF91 model emerges as an exact solution to these equations for the special case of single-residue indels. For the more general case of multiple-residue indels, the equations can be solved by numerical integration. Using simulated data, we show that the resulting distribution over alignments, when compared to previous approximations, is a better fit over a broader range of parameters. We also propose a related approach to develop differential equations for sufficient statistics to estimate the underlying instantaneous indel rates by expectation maximization. Our code and data are available at https://github.com/ihh/trajectory-likelihood.

## Abstract (precis)

How do instantaneous rate models of insertion-deletion processes relate to distributions over pairwise sequence alignments? The only exactly-solved model is the 1991 Thorne.....

IN molecular evolution, the equations of motion describe continuous-time Markov processes on discrete nucleotide or amino acid sequences. For substitution processes, these equations are reasonably well understood, but insertions and deletions (indels) have proved less tractable.

This paper presents a new approach to analysis of indel processes. In this introduction, we first discuss core bioinformatics concepts such as alignments, define a continuous-time Markov process for indels, and review previously published approximations to the finite-time alignment distributions of this process, using hidden Markov models (HMMs). In the remaining sections we describe our new method (in the *Materials and Methods*), report on a simulation-based evaluation (in the *Results*), and discuss the implications of our results (in the *Discussion*).

## Alignments as Summaries of Indel Histories

Our motivating goal is to calculate probabilities of sequence alignments, assuming an underlying instantaneous rate model of indel events. We will mostly consider alignments of two sequences that we will refer to as the “ancestor” and the “descendant,” where the likelihood function takes the form $P\left(\text{descendant},\text{alignment}|\text{ancestor},\text{Θ},t\right)$, where Θ represents model parameters (*e.g.*, mutation rates) and *t* is a time parameter. Common uses of this likelihood function include performing sequence alignment (for downstream inference based on homology, such as protein structure prediction), finding maximum-likelihood estimates of the time parameter *t* (for example, as part of phylogenetic inference of ancestral relationships), and comparing different models or parameterizations Θ (for example, to measure the rate of evolution in sequences, or to annotate conserved regions).

**Figure 1:**
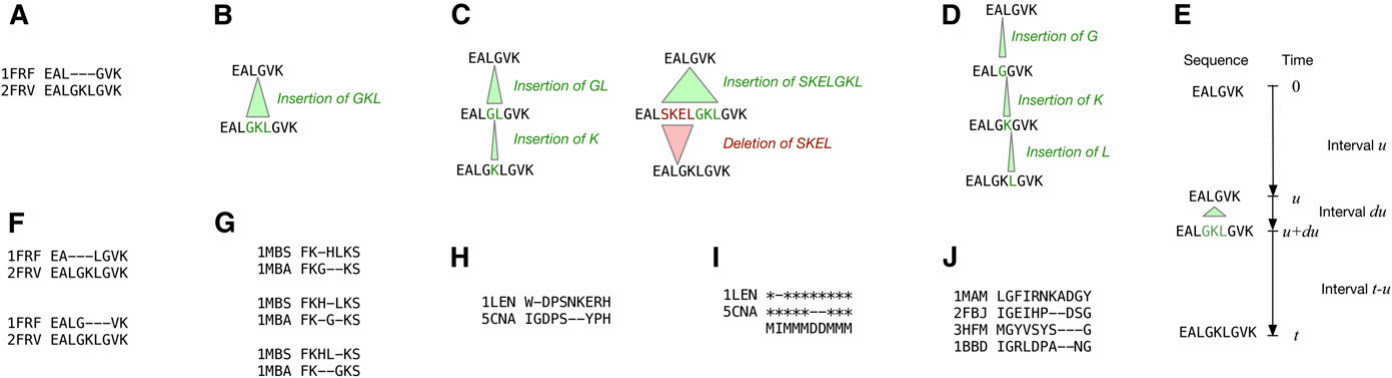
Three views of evolutionary processes—alignments, trajectories, and histories—represent different levels of summarization. Alignments include no information about intermediate events except the positions of homologous residues; trajectories include intermediate sequences and the transition events between them, but not the times at which those events occurred; histories include transition events and times. Panels are illustrated using examples from the HOMSTRAD database (Mizuguchi *et al.* 1998); PDB identifiers are shown. (A) Part of an alignment of two proteins from PDB. (B) A single-event trajectory consistent with alignment A. (C) Two different two-event trajectories consistent with A. (D) A three-event trajectory consistent with A. (E) A history consistent with trajectory (B), in which the single event occurs between times *u* and *u* + *du*. (F) Two alternate alignments of the sequences in alignment A. (G) Several equivalent alignments containing adjacent insertions and deletions that have been rearranged in different ways. (H) An alignment that can only be explained by a model incorporating both substitution and indel events. (I) The gap profile of alignment H, and its associated M/I/D column types. (J) A multiple alignment whose misaligned gap boundaries do not seem to support the TKF92 model’s assumption that multiresidue gaps arise from indivisible sequence fragments.

We seek to derive this pairwise alignment likelihood directly from an instantaneous model of sequence mutation; that is, a continuous-time Markov chain whose state space is the set of all possible DNA or protein sequences. For practical purposes, we often need to summarize paths through this process, and it is worth distinguishing between different ways of doing so. We will use three progressively detailed descriptions of the evolutionary path which we refer to as alignments, trajectories, and histories (Figure 1), as described below.

### Alignments

A pairwise alignment consists of the observed initial and final state of the process (the ancestral and descendant sequences), with gap characters to show which residues are descended from which. An example alignment is shown in Figure 1A. Most of our discussion will be at this level of summarization.

### Trajectories

A trajectory includes all the intermediate sequences from ancestor to descendant. Transitions between the intermediate sequences correspond to instantaneous changes. A trajectory uniquely implies an alignment, but there are many trajectories consistent with each alignment: the most plausible trajectory for alignment 1A is shown in 1B, but the longer trajectories in 1C and 1D are also consistent. We will refer to this level of summarization when discussing some previous methods for indel analysis.

### Histories

A history consists of a trajectory fully annotated with the time of each indel event. This is the most detailed description, being a complete specification of the path of the stochastic process. For each nontrivial trajectory, there is a continuum of possible histories. For example, history 1E is consistent with trajectory 1B, with an event time *u* in the range 0 ≤ *u* ≤ *t*. We will not refer much to this level as it contains more information than we usually care about.

Reflecting this hierarchy of summarization, we can write

$$\begin{array}{l} P\left(\text{descendant}\left|\text{ancestor},\text{ Θ},t\right.\right)=\sum_{\text{alignment}}P\left(\text{descendant},\text{ alignment}\left|\text{ancestor},\text{Θ},t\right.\right)\\ =\sum_{\text{alignment}}\sum_{\text{trajectory}}P\left(\text{descendant},\text{ alignment},\text{ trajectory}\left|\text{ancestor},\text{ Θ},t\right.\right)\\ =\sum_{\text{alignment}}\sum_{\text{trajectory}}\int_{0}^{t}du_{1}\int_{0}^{u_{1}}du_{2}\int_{0}^{u_{2}}du_{3}\ldots P\left(\text{descendant},\text{ alignment},\text{ trajectory},\text{ history}\left|\text{ancestor},\text{Θ},t\right.\right), \end{array}$$

where (*u*_1_, *u*_2,_*u*_3…_) represents all the event times in a history.

Note that the top-level summation is over alignments. Many scenarios demand that we marginalize ambiguous or uncertain alignments. For example, the alignments in 1F are plausible alternatives to 1A; in 1G, the ordering of insertions and deletions may be considered irrelevant for many purposes; and the placement of the second gap in 1H admits some uncertainty.

If the alignment likelihood can be represented as a path probability through a pair HMM, F, then we can perform this sum over alignments using the forward algorithm (Durbin *et al.* 1998), writing the result as

$$\begin{array}{c} F_{XY}=P\left(\text{descendant}=Y\left|\text{ancestor}=X,\text{ Θ},t\right.\right)\\ =\sum_{\varphi }P\left(\text{descendant}=Y,\text{ path}=\varphi \left|\text{ancestor}=X,\text{ Θ},t\right.\right). \end{array}$$

This paper focuses on the probability distribution of alignment gaps. In general, when we refer to a gap, we will mean a run of adjacent indels in any order, as in 1G. Because of the possibility of overlapping indel events, as in 1C, these gaps can arise in a number of different ways.

### The general geometric indel model

Our starting point for defining an evolutionary process is the point substitution model, applied to a sequence. In such a model, each residue evolves according to a substitution rate matrix **R**, such as Kimura’s two-parameter model for DNA (Kimura 1980) or Dayhoff’s PAM model for proteins (Dayhoff *et al.* 1978).

We generalize this by allowing instantaneous insertion and deletion events as well as point substitution events. We do not want to be forced always to count the insertion of multiple adjacent residues as separate events (as in 1D), since this leads to inferential artifacts such as trajectories with too many events, alignments with scattered gaps, rates that are too fast, or times that are too long. Consequently, our model should allow events that insert or delete multiple residues instantaneously (as in 1B), with the indel length being a random variable.

For simplicity, we want to keep the number of parameters minimal, so we specify only the mean lengths of insertion and deletion events. The maximum entropy distribution for this parameterization is the geometric distribution. Thus, the probability that a given event involves *n* residues is $x^{n-1}\left(1-x\right)$ for an insertion, and $y^{n-1}\left(1-y\right)$ for a deletion, with mean lengths $1/\left(1-x\right)$ and $1/\left(1-y\right)$. If the rate of insertions is *λ* and the rate of deletions is *μ,* then, at any given site, an event that inserts *n* residues has rate $\lambda x^{n-1}\left(1-x\right)\times P\left(I_{1}\ldots I_{n}\right)$, where $I_{1}\ldots I_{n}$ represents the actual *n* residues that were inserted, while an event that deletes *n* residues has rate $\mu y^{n-1}\left(1-y\right)$. So, for example, the instantaneous event EALGVK→EAL**GKL**GVK in the history shown in Figure 1E, which occurs during the time interval [*u*, *u* + *du*) and inserts the three residues **GKL**, has instantaneous rate $\lambda x^{2}\left(1-x\right)\times P\left(\text{GKL}\right)$ and infinitesimal probability $\lambda x^{2}\left(1-x\right)P\left(\text{GKL}\right)du$. The inserted residues are independently drawn from the stationary distribution of the substitution model, so $P\left(I_{1}\ldots I_{n}\right)=\prod_{k=1}^{n}\rho_{I_{k}}$ where *ρ***R** = 0. Thus, $P\left(\text{GKL}\right)=\rho_{\text{ G}}\rho_{\text{ K}}\rho_{\text{ L}}$. By contrast, deletion rates are completely independent of the residues being deleted.

To summarize, the parameters of our indel model are \Theta = (\lambda,\mu,x,y,{\bf R}) consisting of indel rates (*λ*, *μ*) and indel length parameters (*x*, *y*), together with a substitution rate matrix **R**. We call this model the general geometric indel (GGI) model, following De Maio (2020). The GGI model is the simplest continuous-time Markov chain over sequences that is local, allows multiresidue indels, and does not enforce reversibility. We may contrast the locality with the Poisson indel process, where the indel rate per site varies inversely with the total sequence length (Bouchard-Côté and Jordan 2013). As for multiresidue indels, other models such as TKF92 do allow this, but they do so by introducing unobservable auxiliary information into the state space; specifically, TKF92 introduces fragment boundaries. We can further constrain the parameters in various ways if desired; for example, by insisting that the model be reversible $\left[\lambda y\left(1-x\right)=\mu x\left(1-y\right)\right]$, as with the long indel model of Miklós *et al.* (2004); or by requiring perfect symmetry between insertions and deletions (*λ* = *μ* and *x* = *y*), as in the simulations of De Maio (2020); or by restricting indels to single residues (*x* = *y* = 0), so all trajectories look like Figure 1D, as with the TKF91 model of Thorne *et al.* (1991).

### Derivation of alignment likelihoods from indel processes

In the previous section, we described the GGI model with instantaneous rates (*λ*,* μ*) and extension probabilities (*x*,*y*). We now review previous approaches to calculating alignment gap likelihoods under this model and related models. These methods include the pair HMMs we evaluate in this paper: TKF91 (Thorne *et al.* 1991), TKF92 (Thorne *et al.* 1992), MLH04 (Miklós *et al.* 2004), LG05 (Löytynoja and Goldman 2005), RS07 (Redelings and Suchard 2007), LAHP19 (Levy Karin *et al.* 2019), and DM20 (De Maio 2020).

All of these methods exploit the property of the GGI model that the indel and substitution processes are independent of one another. A pairwise alignment (1H) has a gap profile (1I) that is like a residue-masked silhouette of the alignment, comprising three types of column: matches (M), in which ancestral and descendant residues are aligned, and insertions (I) and deletions (D), in which either ancestor or descendant contains a gap. We can factorize the alignment likelihood into a term for the gap profile (written as a sequence of M’s, I’s, and D’s) and a conditionally independent set of terms for the actual residue content:

Palignment=W−DPSNKERHIGDPS−−YPH ancestor=WDPSNKERH=Pgap profile=MIMMMDDMMM  length of ancestor=9×MWI×ρG×MDD×MPP×MSS×MEY×MRP×MHH.

Here, *M*_XY_ is the probability that a descendant residue is Y, conditional on the ancestor being X; while *ρ_Y_* is the probability that an inserted residue is Y. Since deletion events are residue-blind in the GGI model, and we have already conditioned on the ancestral sequence, we do not to include terms for the probability that the two deleted ancestral residues are N and K; the gap profile tells us what positions the deleted residues were at, and that is enough.

This decomposition of indel and substitution probabilities is naturally expressed in terms of a pair HMM with M, I, and D states. We can think of the gap profile term as the probability of the state path through the HMM, while the substitution terms correspond to the emission probabilities from those states. The emission part is well understood (Thorne *et al.* 1991): **M** and *ρ* can be linked to an underlying point substitution rate matrix **R** [by the matrix exponential **M** = exp(**R***t*) and the stationary distribution *ρ***R** = 0]. Our focus is on the likelihood of the gap profile: we seek a similar relationship $F\left(t\right)≃\text{exp}\left(\mathbb{R}t\right)$ between the transition probabilities of the pair HMM, $F\left(t\right)$, and the GGI model’s rate matrix over sequences, ℝ.

We now review previous work in this area.

#### TKF91:

The first approach, TKF91, addresses a restricted version of the GGI model allowing only single-residue indels. This reduces to a linear birth-death process, which can be solved exactly (Thorne *et al.* 1991). The probability distribution over alignments can be represented as a pair HMM (Holmes and Bruno 2001). Being exactly solvable, TKF91 has become the canonical example of an indel model. However, as noted previously, it leads to systematic biases during inference, imputing trajectories with too many events, as in Figure 1D.

#### TKF92:

Attempting to address the deficiencies of TKF91, the TKF92 model (Thorne *et al.* 1992) posits a similar birth-death process, but on indivisible multiresidue fragments instead of single residues. Each fragment contains a random, geometrically distributed number of residues. TKF92 has a closed-form pair HMM solution, rather like TKF91, but with the introduction of a new parameter corresponding to the mean fragment length. However, TKF92 is also somewhat unrealistic in practice. The idea that multiresidue gaps arise from unbreakable fragments is artificial, as can be illustrated with reference to the multiple sequence alignment of Figure 1J. The gap boundaries in (1J) do not align, and this is not uncommon: empirically, there is no evidence that TKF92’s indivisible fragments are real. In practice, when using TKF92, it is common to simply marginalize over the fragment boundaries, effectively treating TKF92 as an *ad hoc* approximation to the GGI model. Therefore, by defining a suitable mapping between TKF92’s fragment parameters and the GGI model’s indels, we can evaluate it on this basis, as an approximation to GGI.

#### LG05 and RS07:

Similarly, the LG05 pair HMM used in the PRANK program (Löytynoja and Goldman 2005) and the RS07 pair HMM used in BAliPhy (Redelings and Suchard 2007) introduce fragment length parameters that can be related (with some hand-waving) to the indel length parameters of GGI. In this paper, we evaluate TKF91, TKF92, LG05, and RS07 as approximations to GGI, but we do not evaluate some other indel models that are a little harder to reconcile with GGI because of extra parameters (Rivas and Eddy 2015) or incompatible assumptions (Bouchard-Côté and Jordan 2013).

#### MLH04:

The MLH04 approach, developed by Miklós *et al.* (2004), computes lower-bound likelihoods for alignment gaps by considering short trajectories like those in Figure 1, B–D, integrating out the event times from the corresponding histories to find a likelihood for each such trajectory. To calculate the likelihood of an alignment gap, MLH does a brute-force exhaustive enumeration of all consistent trajectories, up to a given number of indel events and a maximum gap length. Under the assumption of an infinite sequence, the resulting distribution is technically still a pair HMM, albeit one with an infinite number of states (corresponding to every possible size of gap). As our simulations in the *Results* section demonstrate, this approach is extremely slow, and effectively impossible for trajectories with more than three overlapping indel events; however, for very short evolutionary times, MLH04 remains the most accurate approximation to GGI, short of direct simulation.

In the special cases of TKF91 and TKF92, the alignment gap lengths are geometrically distributed. This is not necessarily true in general for the GGI model (Rivas and Eddy 2015): alignment gap lengths are not geometrically distributed even though the underlying indel event lengths are. Thus, a simple three-state pair HMM—whose gap lengths are geometrically distributed—cannot be an exact solution to GGI. Nevertheless, MLH04 shows that the exact solution is, in fact, an infinite-state pair HMM, so a smaller pair HMM may be a reasonable approximation.

#### LAHP19:

A purely simulation-based approach to estimating the gap probabilities of the GGI model has recently been described (Levy Karin *et al.* 2019). In the limit of an infinite number of random trials, this approach is exact. We use such simulations as a gold standard to evaluate other approximations. However, the number of trials required to sample rare outcomes (*i.e.*, long gaps, particularly those involving multiple-event trajectories) is large, and the simulations become computationally expensive with longer sequences. The performance and sampling limitations of this approach are further discussed in the *Results*.

#### DM20:

The DM20 method is a recent breakthrough in approximating the GGI model (De Maio 2020). Starting from the assumption that the alignment likelihood can be approximated by a product of geometric distributions over insertion and deletion lengths, De Maio derived ordinary differential equations (ODEs) for the evolution of the mean lengths of these distributions, yielding transition probabilities for the pair HMM. DM20 is a more accurate approximation to the multiresidue indel process than all previous attempts, although it has limitations: it does not allow deletions to directly follow insertions in the alignment (thus limiting its ability to model covariation between insertion and deletion lengths), it is inexact for the special case of the TKF91 model, and it requires laborious manual derivation of the underlying ODEs.

#### H20:

The H20 method, developed in this paper, builds on DM20 to develop a systematic differential calculus for finding HMM-based approximate solutions of continuous-time Markov processes on strings that are “local” in the sense that the infinitesimal generator is a pair HMM. Our approach addresses the limitations of DM20, identified in the previous paragraph. It does allow deletions to follow insertions, so as to better account for covariation between insertion and deletion gap sizes. The TKF91 model emerges as a special case: the closed-form solutions to TKF91 are also exact solutions to our model. Finally, although our equations can be derived without computational assistance, the analysis is greatly simplified by the use of symbolic algebra packages, both for the manipulation of equations, for which we used Mathematica (Wolfram Research, Inc.) (version 2020), and for the manipulation of state machines, for which we used our recently published software Machine Boss (Silvestre-Ryan *et al.* 2020).

The central idea of our approach is that the application of the infinitesimal generator to the approximating HMM generates a more complicated HMM that, by a suitable coarse-graining operation, can be mapped back to the simpler structure of the approximating HMM. By matching the expected transition usages of these HMMs, we derive ODEs for the transition probabilities of the approximator. Our approach is justified by improved results in simulations, yielding greater accuracy and generality than all previous approaches to this problem, including DM20 (which can be seen as a restricted version of our method). Our approach is further justified by the emergence of the TKF91 model as an exact special case, without the need to introduce any additional latent variables such as fragment boundaries.

While we focus here on the multiresidue indel process, the generality of the infinitesimal automata suggests that other local evolutionary models, such as those allowing neighbor-dependent substitution and indel rates, might also be productively analyzed using this approach.

##### The sequence rate matrix and the infinitesimal-time machine:

We now give a concise preview of the approach described in detail in the *Materials and Methods*.

The rate matrix ℝ of the GGI model is, for two sequences Φ(M→IDN→X):

$${\mathbb{R}}_{XY=}\left\{\begin{array}{l} \sum_{A,C,D}\lambda x^{N-1}\left(1-x\right)\prod_{k=1}^{N}\rho_{C_{k}}\text{ where }X=AD\text{ and }Y=ACD\text{ and }C\in {\text{Ω}}^{N}\\ \sum_{A,B,D}\mu y^{N-1}\left(1-y\right)\text{where}X=ABD\text{ and }Y=AD\text{ and }B\in {\text{Ω}}^{N}\\ \sum_{A,B,C,D}R_{BC}\text{where}X=ABD\text{ and }Y=ACD\text{ and }B,C\in \text{Ω},B\neq C\\ -\sum_{Z\neq X}{\mathbb{R}}_{XZ}\text{where}X=Y\text{ and }Z\in \Omega^{*} \end{array}\right..$$

where Ω is the residue alphabet (*e.g.*, nucleotides or amino acids), Ω* is the set of all sequences over that alphabet (including the empty sequence *ε*), Ω*^N^* is the set of all sequences of finite length *N*, *B* is the deleted sequence, *C* is the inserted sequence, and $A,D\in \Omega^{*}$ are flanking sequences (we will mostly be considering the infinite-sequence approximation, where X,Y,A,D≠ε).

**Figure 2:**
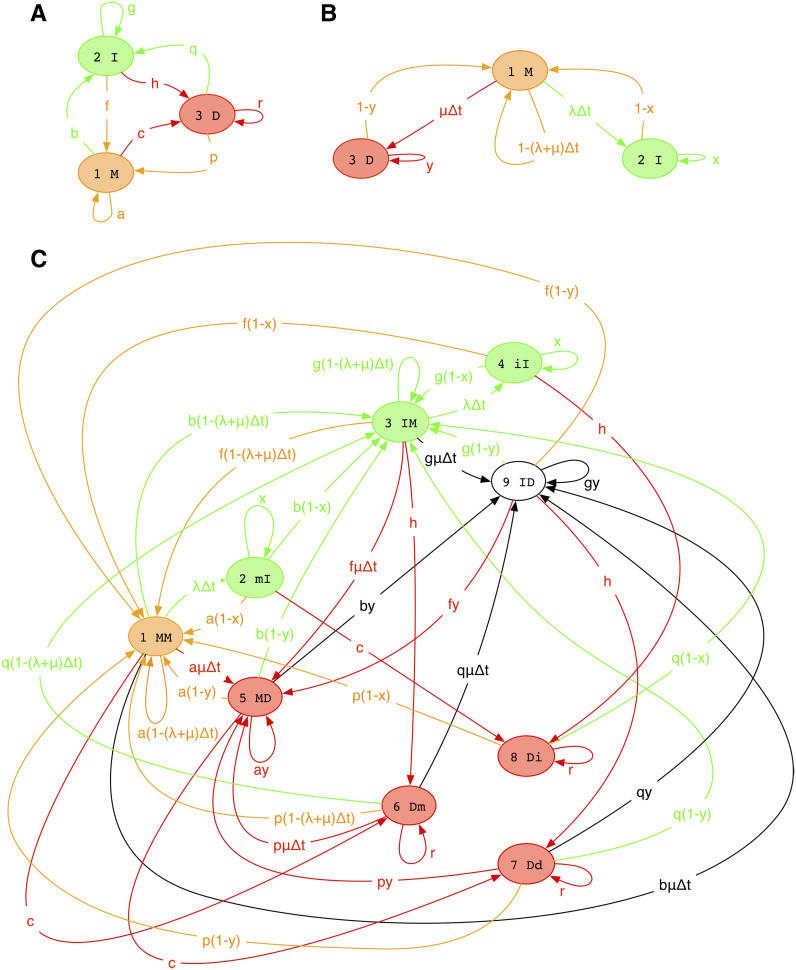
Three state machines for modeling indels in alignments. Match states (*σ*_M_) are orange, insert states (*σ*_I_) are green, delete states (*σ*_D_) are red, and null states (*σ*_N_) are uncolored. Transitions are colored by destination state. States for the machines in A–C are further described in Tables 1, 3, and 4. Our approach is to approximate C with a machine of the same form as A. (A) Machine $F\left(t\right)$, defined under *Three-state HMM* and in Table 3, models alignments at divergence time *t*. (B) Machine $G\left(\text{Δ}t\right)$, defined under *Infinitesimal-time machine* and in Table 1, models the infinitesimal evolution over time Δ*t*. (C) Machine $F\left(t\right)G\left(\text{Δ}t\right)$, defined under *Rate of change of expected transition counts* and in Table 4, models alignments at divergence time *t* + Δ*t*. It is the machine product of $F\left(t\right)$ and $G\left(\text{Δ}t\right)$: each state has the form XY where X is an F state and Y is a G state. Uppercase is used to indicate that a component machine makes a transition when the compound state is entered. So, for example, when FG makes the transition mI → MM, the transition weight is the product of *a* (for F’s self-looping M → M transition) and 1 − *x* (for G’s I → M transition). However, if then makes the transition MM → Dm, the transition weight is just *c* (for F’s M → D transition), since G stays in the M state without making a transition. This structure arises from simple rules for transition synchronization in multiplied machines (Westesson *et al.* 2011, 2012).

Suppose that $\psi \left(t\right)\in \Omega^{*}$ is a sequence evolving under the GGI model. Consider $G\left(\text{Δ}t\right)$, the pair HMM defined in Figure 2B and Table 1. Assuming *ψ* (*t*) is infinitely long, the forward algorithm for $G\left(\text{Δ}t\right)$ computes the conditional distribution over an instant of evolutionary time:

**Table 1:** Interpretation of states in machine $G\left(\text{Δ}t\right)$ (Figure 2B, defined in *Infinitesimal-time machine*); here, $\omega_{\text{ in}},\omega_{\text{ out}}\in \text{Ω}$ represent input and output tokens from the residue alphabet

State	Name	Class	On entry	Input	Output	*P* (*ω*_out_)
1	M	*σ*_M_	Reads *ω*_in_ from input, writes *ω*_out_ to output	*ω*_in_	*ω*_out_	$\exp{\left(Rt\right)}_{\omega_{\text{in}}\omega_{\text{out}}}$
2	I	*σ*_I_	Writes *ω*_out_ to output	—	*ω*_out_	$\rho_{\omega_{\text{out}}}$
3	D	*σ*_D_	Reads *ω*_in_ from input	*ω*_in_	—	—

$$\begin{array}{c} G{\left(\text{Δ}t\right)}_{XY}=P\left(\psi (t+\text{Δ}t)=Y\left|\psi \left(t\right)=X,\text{Θ},t\right.\right)+o\left(\text{Δ}t\right)\\ =\text{exp}{\left(\mathbb{R}\text{Δ}t\right)}_{XY}+o\left(\text{Δ}t\right)\\ =I+{\mathbb{R}}_{XY}\text{Δ}t+o\left(\text{Δ}t\right), \end{array}$$

where I is the identity matrix over sequences ($I_{XY}=1$ if *X* = *Y*, 0 if *X* ≠ *Y*).

Our approach is to find a pair HMM, $F\left(t\right)$ (Figure 2A), that approximates the matrix exponential $F\left(t\right)≃\text{exp}\left(\mathbb{R}t\right)={\text{lim}}_{\text{Δ}t\to 0}{\left(G\left(\text{Δ}t\right)\right)}^{t/\text{Δ}t}$, by mapping the machine product $F\left(t\right)G\left(\text{Δ}t\right)$ (Figure 2C) back onto $F\left(t+\text{Δ}t\right)$. We match expected transition counts between classes of states in $F\left(t\right)G\left(\text{Δ}t\right)$ to their representative transitions in $F\left(t+\text{Δ}t\right)$, and take the limit Δ*t* → 0 to derive differential equations for the transition weights of $A\text{'}=Q^{\left(\text{MIDN}\to \text{Y}\right)}$.

**Figure 3:**
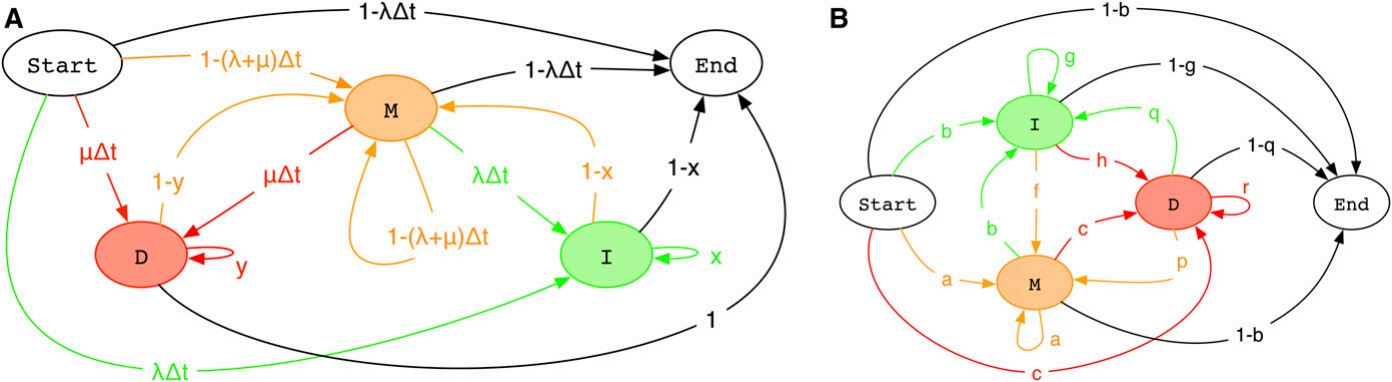
Versions of the first two pair HMMs of Figure 2 that include start and end states, and so can be used for finite sequences. As in Figure 2, match states (*σ*_M_) are orange, insert states (*σ*_I_) are green, delete states (*σ*_D_) are red, and null states (*σ*_N_) are uncolored. (A) A version of \mathbb{G}(\Delta t) with start and end states, where deletion rates at the end of the sequence are elevated by a factor 1/(1-y) so that the total rightward deletion rate at any residue is \mu, independent of its distance from the end. (B) A version of \mathbb{F}(t) with start and end states.

A note on finite sequences: to model these we can set ${\mathbb{R}}_{AB,A}=\mu y^{N-1}$ for $B\in {\text{Ω}}^{N}$, dropping the 1 − *y* term for deletions that remove the end of the sequence. This ensures the total rightward deletion rate starting at any residue is *μ*, regardless of its distance from the end. We can then define $G\left(\text{Δ}t\right)$ as in Figure 3A. Imposing reversibility on finite-sequence models takes slightly more care (Miklós *et al.* 2004).

## Materials and Methods

As noted in the *Introduction*, our approach makes use of pair HMMs. We assume some familiarity with these models, which are standard in bioinformatics. A tutorial introduction can be found in Durbin *et al.* (1998).

The pair HMMs we will use are normalized for the conditional probability $P\left(\text{descendant}\left|\text{ancestor}\right.\right)$ [rather than for the joint distribution $P\left(\text{ancestor},\text{descendant}\right)$ as is often seen in the bioinformatics literature]. Such conditionally normalized pair HMMs are sometimes called input-output automata, or transducers. Rather than simultaneously generating two output sequences, these state machines read a specified input sequence and generate a probabilistic output. Following the convention of Durbin *et al.* (1998), these pair HMMs have Match (input-output), Insert (output-only), and Delete (input-only) states corresponding to the M, I, and D columns in a pairwise alignment (see *e.g.*, Figure 1I), as well as Null (N) states that do not input or output anything.

A key result enabling our approach is that two automata $\left(A,B\right)$ can be multiplied together: the output of A is fed into the input of B. The serial operation of both machines can be represented by a single compound machine AB, constructed algorithmically from the two component machines. The algorithm takes a Cartesian product of the two machines’ state spaces, then synchronizes transitions in this joint space so that each output-writing transition of A coincides with an input-reading transition of B, with some ordering of updates so that indels are not double-counted. The algorithms for doing these multiplications are published (Westesson *et al.* 2011, 2012), and software implementations are available (Silvestre-Ryan *et al.* 2020).

For our purposes, the only machine-multiplication that we need is the one shown in Figure 2. A three-state machine representing a distribution over finite-time pairwise alignments [$F\left(t\right)$, Figure 2A] is multiplied by another three-state machine representing the action of the GGI model over an infinitesimal time interval [$G\left(\text{Δ}t\right)$, Figure 2B], yielding a nine-state machine [$F\left(t\right)G\left(\text{Δ}t\right)$, Figure 2C].

In the following sections, we show that $F\left(t\right)G\left(\text{Δ}t\right)$ can be systematically mapped back to $F\left(t+\text{Δ}t\right)$ by a coarse-graining operation that involves finding the expected number of transitions of each type (I→I, I→D, *etc*.) in walks through the state space that begin and end in the M state. In the following sections, we describe how to calculate these expectations, with expository examples relating to $F\left(t\right)$, which we then define in detail. We then apply this to map $F\left(t\right)G\left(\text{Δ}t\right)$ back to $F\left(t+\text{Δ}t\right)$, and, by taking the limit Δ*t* → 0, derive differential equations for the transition probabilities of $F\left(t\right)$. Finally, we show that this reduces correctly to the TKF91 model when *x* = *y* = 0.

Table 2 gives a glossary of mathematical terms used throughout this section. In the Supplemental Material, we give some additional lemmas and a conjecture relating this work to the expectation-maximization algorithm for parameter estimation.

**Table 2:** Glossary of mathematical notation, terminology, and abbreviations used in this paper

Term	Meaning	Defined in
History	Realization of a continuous-time process, including event times	*Introduction*, Figure 1
Trajectory	Summary of a history that includes only events but not times	*Introduction*, Figure 1
Alignment	Summary of a trajectory that shows homologous residues	*Introduction*, Figure 1
Pair HMM	A hidden Markov model for pairwise alignments	Durbin *et al.* (1998)
GGI	The general geometric indel model	*Introduction*
TKF91	The links model, a special case of GGI for single-residue indels	Thorne *et al.* (1991)
TKF92	Sequel to the TKF91 model allowing multiresidue indels	Thorne *et al.* (1992)
MLH04	Approximation to GGI that enumerates short trajectories	Miklós *et al.* (2004)
LG05	Pair HMM used by PRANK alignment software	Löytynoja and Goldman (2005)
RS07	Pair HMM used by BAliPhy alignment software	Redelings and Suchard (2007)
LAHP19	Approximation to GGI that estimates gap probabilities by simulation	Levy Karin *et al.* (2019)
DM20	The cumulative indel model, a direct precursor to this work	De Maio (2020)
*λ*, *μ*	Rate of beginning an insertion or deletion in the GGI model	*Introduction*
*x*, *y*	Probability of extending an insertion or deletion in the GGI model	*Introduction*
**R**	Substitution rate matrix in the GGI model	*Introduction*
Θ	The set of GGI model parameters (*λ*, *μ*, *x*, *y*, **R**)	*Introduction*
*t*	Evolutionary time separating ancestor and descendant sequences	*Introduction*
Δ*t*	A very small amount of evolutionary time	*Introduction*
**M**	Substitution probability matrix defined by **M** = exp (**R***t*)	*Introduction*
*ρ*	Probability distribution over inserted residues defined by *ρ***R** = 0	*Introduction*
Ω	The residue alphabet, *e.g.*, nucleotides or amino acids	*Introduction*
Ω*^N^*,Ω*	Sets of sequences over Ω (*N* denotes fixed length, * denotes any length)	*Introduction*
*Ε*	The empty sequence	*Introduction*
ℝ	The rate matrix over Ω* for the GGI model	*Introduction*
M	A generic pair HMM, a probabilistic input-output machine	*Materials and Methods*
*K*	Number of states in the machine	*Materials and Methods*
*σ*_M_, *σ*_I_, *σ*_D_, *σ*_N_	Sets of Match-, Insert-, Delete-, and Null-type states	*Materials and Methods*
*σ*_IDN_, *σ*_MIDN_, etc.	Unions of state classes, *e.g.*, $\sigma_{\text{IDN}}=\sigma_{\text{I}}\cup^{\text{​}}\sigma_{\text{D}}\cup^{\text{​}}\sigma_{\text{N}}$	*Materials and Methods*
*φ*	A state path through a pair HMM	*Materials and Methods*
**Q**	Transition probability matrix for a pair HMM	*Materials and Methods*
$Q\left[M\right]$	Transition matrix for a particular machine M	*Materials and Methods*
$\text{Φ}\left(\text{X}\to \text{Y}\to \text{Z}\right)$	The set of all paths starting in *σ*_X_, possibly passing through states in *σ*_Y_, and ending in *σ*_Z_	*Materials and Methods*
$\text{Φ}\left(\text{M}\to \text{IDN}\to \text{M}\right)$	The set of all paths that begin and end in the Match state, but do not otherwise use it for the intervening steps	*Materials and Methods*
$J^{\left(\text{X}\to \text{Y}\right)}$	A matrix that contains 1’s for entries corresponding to *σ*_X_ → *σ*_Y_ transitions, and 0’s for other entries	*Materials and Methods*
$Q^{\left(\text{X}\to \text{Y}\right)}$	A matrix that contains probabilities for *σ*_X_ → *σ*_Y_ transitions, and 0’s for other entries	*Materials and Methods*
○	The pointwise matrix product, defined by ${\left(A\circ B\right)}_{ij}\equiv A_{ij}B_{ij}$	*Materials and Methods*
$S_{\text{X}}\left(\varphi \right)$	The number of *σ*_X_ states in path *φ*	*Materials and Methods*
$T_{\text{XY}}\left(\varphi \right)$	The number of *σ*_X_ → *σ*_Y_ transitions in path *φ*	*Materials and Methods*
$E_{\varphi \left|M\right.}\left[\ldots \right]$	An expectation over $\text{Φ}\left(\text{M}\to \text{IDN}\to \text{M}\right)$ for machine, M.	*Materials and Methods*
**U**, **V**, **W**	Geometric series sums involving the transition matrix **Q**	Equation 1
$F\left(t\right)$	A pair HMM approximation to finite-time alignments under the GGI model. Sometimes abbreviated to F	Figure 2A and 1
*a*, *b*, *c*, *f*, *g*, *h*, *p*, *q*, *r*	Transition probabilities of $F\left(t\right)$. All are functions of *t*	Equation 3 and Equation 4
${\bar{S}}_{\text{X}}$	Expectation of *S*_X_ over $\text{Φ}\left(\text{M}\to \text{IDN}\to \text{M}\right)$	Equation 2, Equation 4, and Equation 7
${\bar{T}}_{\text{XY}}$	Expectation of *T*_XY_ over $\text{Φ}\left(\text{M}\to \text{IDN}\to \text{M}\right)$	Equation 2, Equation 5, and Equation 6
$G\left(\text{Δ}t\right)$	A pair HMM for alignments at infinitesimal time intervals under the GGI model. Sometimes abbreviated to G	Figure 2B and Table 1
$F\left(t\right)G\left(\text{Δ}t\right)$	Automata product of $F\left(t\right)$ and $G\left(\text{Δ}t\right)$. Abbreviated to FG	Figure 2C and Table 4
0	A *K* × *K* matrix of zeroes	
1	A *K* × *K* matrix of ones	

### Expected transition usage

Suppose that we have a pair HMM, M, with *K* states that can be partitioned into match *σ*_M_, insert *σ*_I_, delete *σ*_D_, and null *σ*_N_ states. As a shorthand we will write *σ*_ID_ for $\sigma_{I}\cup \sigma_{D}$, and so on. Thus *σ*_MIDN_ is the complete set of *K* states.

We will be considering models with only one match state, which, by convention, will always be the first state, so $\sigma_{M}=\left\{1\right\}$

Let $\varphi =\left(\varphi_{1}\ldots \varphi_{n}\right)$ denote a state path. The transition probability matrix is **Q** with elements $Q_{ij}=P\left(\varphi_{k+1}=j\left|\varphi_{k}=i\right.\right)$.

For $\text{X, Y, Z}\subseteq \left\{\text{M, I, D, N}\right\}$, let $\text{Φ}\left(\text{X}\to \text{Y}\to \text{Z}\right)$ denote the set of state paths with the following properties:

The path begins in an *σ_X_* state;
The path ends in an *σ_Z_* state;
For paths with more than just a begin and end state, the intermediate states are all *σ*_Y_ states.

Let $J^{\left(\text{X}\to \text{Y}\right)}$ be a matrix that selects transitions from *σ*_X_ to *σ*_Y,_

$$J_{ij}^{\left(\text{X}\to \text{Y}\right)}=\left\{\begin{array}{ll} 1 & i\in \sigma_{\text{X}},j\in \sigma_{\text{Y}}\\ 0 & \text{otherwise} \end{array}\right.,$$

and let $Q^{\left(\text{X}\to \text{Y}\right)}\equiv J^{\left(\text{X}\to \text{Y}\right)}\circ Q$, where ∘ is the pointwise product, defined as follows: if *A*, *B* are two matrices of the same size, then ${\left(A\circ B\right)}_{ij}\equiv A_{ij}B_{ij}$.

A concrete example is the machine $F\left(t\right)$ in Figure 2A, which has *σ*_M_ = {1}, *σ*_I_ = {2}, *σ*_D_ = {3}, and $\sigma_{\text{N}}=\emptyset$. Thus, for example, *σ*_MIDN_ = {1, 2, 3} and *σ*_IDN_ = {2,3}. Some examples of state paths in $\text{Φ}\left(\text{M}\to \text{IDN}\to \text{M}\right)$ are $\left(1,1\right)$, $\left(1,2,2,2,1\right)$, and $\left(1,3,3,2,3,1\right)$.

The transition matrix is $Q=\left(\begin{array}{ccc} a & b & c\\ f & g & h\\ p & q & r \end{array}\right)$. The matrix $J^{\left(\text{MID}\to \text{ID}\right)}=\left(\begin{array}{ccc} 0 & 1 & 1\\ 0 & 1 & 1\\ 0 & 1 & 1 \end{array}\right)$ selects transitions into the *I* and *D* states, so $Q^{\left(\text{MID}\to \text{ID}\right)}=\left(\begin{array}{ccc} 0 & b & c\\ 0 & g & h\\ 0 & q & r \end{array}\right)$.

Continuing with the example of machine $F\left(t\right)$ in Figure 2A, the state path (1, 2, 2, 2, 1) has state types (M, I, I, I, M_)_ and thus transitions (MI, II, II, IM), so the transition counts are *T*_MI_ = *T*_IM_ = 1 and *T*_II_ = 2. For an example of a machine with more complex structure including null states, consider $F\left(t\right)G\left(\text{Δ}t\right)$ of Figure 2C. A state path $\left(1,2,3,9,9,5,1\right)$ through this machine has state types (M, I, I, N, N, D, M). When we remove the null states, this becomes (M, I, I, D, M) and so the transitions are (MI, II, ID, DM). Thus the transition counts are $T_{\text{MI}}=T_{\text{II}}=T_{\text{ID}}=T_{\text{DM}}=1$.

Consider a random walk $\varphi \in \text{Φ}\left(M\to IDN\to M\right)$ that begins and ends in the match state, passing only through nonmatch states in between. Let $T_{\text{XY}}\left(\varphi \right)=\left|\left\{\left(i,j\right):j>i,\left(\varphi_{i}\ldots \varphi_{j}\right)\in \text{Φ}\left(\text{X}\to \text{N}\to \text{Y}\right)\right\}\right|$ count the number of transitions from X states to Y states if null states are removed from the walk. In other words this is the number of subpaths of *φ* that go from a state in *σ*_X_, via zero or more null states, to a state in *σ*_Y_.

To find the expected value of *T*_XY_ in walks that begin and end in the match state, we break down paths into three segments: M to X (via insert, delete, and null states), X to Y (via null states), and Y to M (via insert, delete, and null states). The first and last segments are only required if M ≠ X and Y ≠ M. The corresponding sets of state paths are $\text{Φ}\left(\text{M}\to \text{IDN}\to \text{X}\right)$, $\text{Φ}\left(\text{X}\to \text{N}\to \text{Y}\right)$, and $\text{Φ}\left(\text{Y}\to \text{IDN}\to \text{M}\right)$.

To sum over all paths in a given set $\text{Φ}\left(\text{X}\to \text{Y}\to \text{Z}\right)$, we can use the geometric series formula $B=\sum_{k=0}^{\infty }A^{k}={\left(1-A\right)}^{-1}$, where **1** is the *K* × *K* identity matrix. Setting $A=Q^{\left(\text{Y}\to \text{Y}\right)}$, the effective X → Z transition probabilities are the nonzero entries of $C=Q^{\left(\text{X}\to \text{Z}\right)}+Q^{\left(\text{X}\to \text{Y}\right)}BQ^{\left(\text{Y}\to \text{Z}\right)}$. We can further simplify the formulae, for example by noting that $C=J^{\left(\text{X}\to \text{Z}\right)}\circ \left(Q+A\prime BQ\right)=J^{\left(\text{X}\to \text{Z}\right)}\circ \left(B\prime Q\right)$ where $A\prime =Q^{\left(\text{MIDN}\to \text{Y}\right)}$ and $B\prime ={\left(1-A\prime \right)}^{-1}$.

Using the methods of the previous paragraphs, the expectation of *T_XY_* is

$$\begin{array}{c} E_{\varphi |M}\left[T_{\text{XY}}\left(\varphi \right)\right]\\ =\sum_{\varphi \in \text{Φ}\left(\text{M}\to \text{IDN}\to \text{M}\right)}P\left(\varphi \right)T_{XY}\left(\varphi \right)\\ ={\left(\sum_{i=0}^{\infty }{\left(Q^{\left(\text{MIDN}\to \text{IDN}\right)}\right)}^{i}\left(J^{\left(\text{X}\to \text{Y}\right)}\circ \sum_{j=0}^{\infty }{\left(Q^{\left(\text{MIDN}\to \text{N}\right)}\right)}^{j}Q\right)\sum_{k=0}^{\infty }{\left(Q^{\left(\text{IDN}\to \text{MIDN}\right)}\right)}^{k}\right)}_{11}\\ ={\left(U\left(J^{\left(\text{X}\to \text{Y}\right)}\circ \left(VQ\right)\right)W\right)}_{11}, \end{array}$$ (1)

where

$$\begin{array}{c} U={\left(1-Q^{\left(\text{MIDN}\to \text{IDN}\right)}\right)}^{-1}\\ V={\left(1-Q^{\left(\text{MIDN}\to \text{N}\right)}\right)}^{-1}\\ W={\left(1-Q^{\left(\text{IDN}\to \text{MIDN}\right)}\right)}^{-1}. \end{array}$$

Let $S_{X}\left(\varphi \right)$ be the number of X states in *φ*, excluding the final state. Thus,

$$S_{M}\left(\varphi \right)=1$$

$$\begin{array}{c} S_{X}\left(\varphi \right)=\sum_{\text{Y}\in \text{M,I,D,N}}T_{\text{XY}}\left(\varphi \right)\\ =\sum_{\text{Y}\in \text{M,I,D,N}}T_{\text{YX}}\left(\varphi \right). \end{array}$$

### Three-state HMM

Consider the machine $F\left(t\right)$ shown in Figure 2A with $\sigma_{\text{M}}=\left\{1\right\}$, $\sigma_{I}=\left\{2\right\}$, $\sigma_{\text{D}}=\left\{3\right\}$, and

$$Q\left[F\left(t\right)\right]=\left(\begin{array}{ccc} a\left(t\right) & b\left(t\right) & c\left(t\right)\\ f\left(t\right) & g\left(t\right) & h\left(t\right)\\ p\left(t\right) & q\left(t\right) & r\left(t\right) \end{array}\right),$$

with *a* + *b* + *c* = 1, *f* + *g* + *h*, and *p* + *q* + *r* = 1. Table 3 gives the emission probabilities.

**Table 3:** Interpretation of states in machine $F\left(t\right)$ (Figure 2A, defined in *Three-state HMM*); here, $\omega_{\text{ in}},\omega_{\text{ out}}\in \text{Ω}$ represent input and output tokens from the residue alphabet

State	Name	Class	On entry	Input	Output	*P* (*ω*_out_)
1	M	*σ*_M_	Reads *ω*_in_ from input, writes *ω*_out_ to output	*ω*_in_	*ω*_out_	$\text{exp}{\left(R\Delta t\right)}_{\omega_{\text{in}}\omega_{\text{out}}}$
2	I	*σ*_I_	Writes *ω*_out_ to output	—	*ω*_out_	$\rho_{\omega_{\text{out}}}$
3	D	*σ*_D_	Reads *ω*_in_ from input	*ω*_in_	—	—

Here, *t* will play the role of a time parameter.

Let

$$\begin{array}{c} {\bar{T}}_{\text{XY}}\left(t\right)=E_{\varphi |F\left(t\right)}[T_{\text{XY}}\left(\varphi \right)]\\ {\bar{S}}_{\text{X}}\left(t\right)=E_{\varphi |F\left(t\right)}[S_{\text{X}}\left(\varphi \right)]=\sum_{\text{Y}\in \text{M,I,D,N}}{\bar{T}}_{\text{XY}}\left(t\right)=\sum_{\text{Y}\in \text{M,I,D,N}}{\bar{T}}_{\text{YX}}\left(t\right)\\ {\bar{S}}_{\text{M}}\left(t\right)=1, \end{array}$$ (2)

where the expectations are as defined in (1) [throughout this paper, such expectations are over $\varphi \in \text{Φ}\left(M\to IDN\to M\right)$]. Evidently,

$$\begin{array}{l} a\left(t\right)={\bar{T}}_{\text{MM}}\left(t\right),b\left(t\right)={\bar{T}}_{\text{MI}}\left(t\right),c\left(t\right)={\bar{T}}_{\text{MD}}\left(t\right),\\ f\left(t\right)={\bar{T}}_{\text{IM}}\left(t\right)/{\bar{S}}_{\text{I}}\left(t\right),g\left(t\right)={\bar{T}}_{\text{II}}\left(t\right)/{\bar{S}}_{\text{I}}\left(t\right),h\left(t\right)={\bar{T}}_{\text{ID}}\left(t\right)/{\bar{S}}_{\text{I}}\left(t\right),\\ p\left(t\right)={\bar{T}}_{\text{DM}}\left(t\right)/{\bar{S}}_{\text{D}}\left(t\right),q\left(t\right)={\bar{T}}_{\text{DI}}\left(t\right)/{\bar{S}}_{\text{D}}\left(t\right),r\left(t\right)={\bar{T}}_{\text{DD}}\left(t\right)/{\bar{S}}_{\text{D}}\left(t\right). \end{array}$$ (3)

By (1),

$$\begin{array}{c} {\bar{S}}_{I}\left(t\right)=\frac{\left(b\left(1-r\right)+cq\right)\left(f\left(1-r\right)+hp\right)}{{\left(\left(1-g\right)\left(1-r\right)-hg\right)}^{2}}\\ {\bar{S}}_{D}\left(t\right)=\frac{\left(c\left(1-g\right)+bh\right)\left(p\left(1-g\right)+fq\right)}{{\left(\left(1-g\right)\left(1-r\right)-hq\right)}^{2}}. \end{array}$$ (4)

The essence of our approach is to use transducer composition to study infinitesimal increments in ${\bar{T}}_{\text{XY}}\left(t\right)$, and thereby obtain differential equations that can be solved to find these parameters.

### Infinitesimal-time machine

The infinitesimal transducer $G\left(\text{Δ}t\right)$ of Figure 2B has states $\sigma_{M}=\left\{1\right\}$, $\sigma_{I}=\left\{2\right\}$, $\sigma_{D}=\left\{3\right\}$, and transition matrix

$$Q\left[G\left(\text{Δ}t\right)\right]=\left(\begin{array}{ccc} 1-\left(\lambda +\mu \right)\text{Δ}t & \lambda \text{Δ}t & \mu \text{Δ}t\\ 1-x & x & 0\\ 1-y & 0 & y \end{array}\right).$$

See Table 1 for emission probabilities. This describes the GGI model as defined in the introduction. The model parameters are the insertion and deletion rates (*λ*, *μ*) and extension probabilities (*x*, *y*), and the time interval $\text{Δ}t\ll 1/\left(\lambda +\mu \right)$.

### Rate of change of expected transition counts

Composing $F\left(t\right)$ (Figure 2A) with $G\left(\text{Δ}t\right)$ (Figure 2B) yields $F\left(t\right)G\left(\text{Δ}t\right)$, the machine of Figure 2C.

This machine has states $\sigma_{\text{M}}=\left\{1\right\}$, $\sigma_{\text{I}}=\left\{2,3,4\right\}$, $\sigma_{\text{D}}=\left\{5,6,7,8\right\}$, $\sigma_{\text{N}}=\left\{9\right\}$, and transition matrix

$$Q[F\left(t\right)G\left(\text{Δ}t\right)]=\left(\begin{array}{ccccccccc} a\left(1-\left(\lambda +\mu \right)\text{Δ}t\right) & \lambda \text{Δ}t & b\left(1-\left(\lambda +\mu \right)\text{Δ}t\right) & 0 & a\mu \text{Δ}t & c & 0 & 0 & b\mu \text{Δ}t\\ a\left(1-x\right) & x & b\left(1-x\right) & 0 & 0 & 0 & 0 & c & 0\\ f\left(1-\left(\lambda +\mu \right)\text{Δ}t\right) & 0 & g\left(1-\left(\lambda +\mu \right)\text{Δ}t\right) & \lambda \text{Δ}t & f\mu \text{Δ}t & h & 0 & 0 & g\mu \text{Δ}t\\ f\left(1-x\right) & 0 & g\left(1-x\right) & x & 0 & 0 & 0 & h & 0\\ a\left(1-y\right) & 0 & b\left(1-y\right) & 0 & ay & 0 & c & 0 & by\\ p\left(1-\left(\lambda +\mu \right)\text{Δ}t\right) & 0 & q\left(1-\left(\lambda +\mu \right)\text{Δ}t\right) & 0 & p\mu \text{Δ}t & r & 0 & 0 & q\mu \text{Δ}t\\ p\left(1-y\right) & 0 & q\left(1-y\right) & 0 & py & 0 & r & 0 & qy\\ p\left(1-x\right) & 0 & q\left(1-x\right) & 0 & 0 & 0 & 0 & r & 0\\ f\left(1-y\right) & 0 & g\left(1-y\right) & 0 & fy & 0 & h & 0 & gy \end{array}\right).$$

Table 4 describes the interpretation of each state, and its emission probability distribution.

**Table 4:** Interpretation of states in the composite machine $F\left(t\right)G\left(\text{Δ}t\right)$ (Figure 2C, defined in *Rate of change of expected transition counts*)

State	Name	Class	On entry	Input	Output	*P* (*ω*_out_)
1	MM	*σ*_M_	F reads input *ω*_in_, writes *ω*_thru_ to G, enters M state	*ω*_in_	*ω*_out_	$\text{exp}{\left(R\left(t+\text{Δ}t\right)\right)}_{\omega_{\text{in}}\omega_{\text{out}}}$
			G reads *ω*_thru_ from F, writes output *ω*_out_, enters M state			
2	mI	*σ*_I_	F stays in M state (no transition)	—	*ω*_out_	$\rho_{\omega_{\text{out}}}$
			G writes output *ω*_out_, enters I state			
3	IM	*σ*_I_	F writes *ω*_thru_ to G, enters I state	—	*ω*_out_	$\rho_{\omega_{\text{out}}}$
			G reads *ω*_thru_ from F, writes output *ω*_out_, enters M state			
4	iI	*σ*_I_	F stays in I state (no transition)	—	*ω*_out_	$\rho_{\omega_{\text{out}}}$
			G writes output *ω*_out_, enters I state			
5	MD	*σ*_D_	F reads input *ω*_in_, writes *ω*_thru_ to G, enters M state	*ω*_in_	—	—
			G reads *ω*_thru_ from F, enters D state			
6	Dm	*σ*_D_	F reads input *ω*_in_, enters D state	*ω*_in_	—	—
			G stays in M state (no transition)			
7	Dd	*σ*_D_	F reads input *ω*_in_, enters D state	*ω*_in_	—	—
			G stays in D state (no transition)			
8	Di	*σ*_D_	F reads input *ω*_in_, enters D state	*ω*_in_	—	—
			G stays in I state (no transition)			
9	ID	*σ*_D_	F writes *ω*_thru_ to G, enters I state	—	—	—
			G reads *ω*_thru_ from F, enters D state			

Here, $\omega_{\text{ in}},\omega_{\text{ out}},\omega_{\text{ thru}}\in \text{Ω}$ represent input, output, and pass-through tokens from the residue alphabet. Each state has the form XY where X is an F state and Y is a Gstate. Each transition of FG can involve an F-transition, a G-transition, or both. Uppercase (XY) is used to indicate that a component machine makes a transition when the compound state is entered; lowercase (xy) indicates the component machine makes no transition. Thus, transitions into $\left\{\text{MM, IM, MD, Dm, Dd, Di, ID}\right\}$ involve an F-transition; transitions into $\left\{\text{MM, mI, IM, iI, MD, ID}\right\}$ involve a G-transition. This structure arises from simple rules for transition synchronization in multiplied machines (Westesson *et al.* 2011, 2012). By these rules, G can only make an input-reading transition when F makes an output-writing transition, and vice versa. So, for example, when FG makes the transition mI → MM, what happens internally is that F makes a self-looping M → M transition while G makes an I → M transition, and an (unobserved) token *ω*_thru_ is passed through from F to G. However, if FG then makes the transition MM → Dm, internally F makes a M → D transition without outputting anything, so G just stays in the M state without making a transition.

The transducer composition $F\left(t\right)\times G\left(\text{Δ}t\right)$ was performed using the automata algebra program Machine Boss (Silvestre-Ryan *et al.* 2020). A general procedure for doing this for any two machines A,B involves taking the Cartesian product of the two machines’ state spaces and then synchronizing their transitions so that the output of A drives the input of B. This ensures that, if $M_{XY}$ represents the result of the forward algorithm for machine M (with null states eliminated) and sequences X,Y, then ${\left(AB\right)}_{XZ}=\sum_{Y}A_{XY}B_{YZ}$. More details on these operations can be found elsewhere (Silvestre-Ryan *et al.* 2020; Westesson *et al.* 2011, 2012) and their information-theoretic and linguistic roots in Mohri *et al.* (2002).

We now make the approximation $F\left(t\right)G\left(\text{Δ}t\right)\approx F\left(t+\text{Δ}t\right)$, which is to say that the nine-state machine of Figure 2C can be approximated by the three-state machine of Figure 2A by infinitesimally increasing the time parameter of the simpler machine. This will not, in general, be exact (with the exception of the TKF91 model, discussed in the next section). However, by mapping states (and hence transitions) of FG back to F, and setting F’s transition probabilities proportional to the expected number of times the corresponding transitions are used in FG, we find a maximum-likelihood fit.

The expected transition counts evolve via the coupled differential equations

$$\begin{array}{c} \frac{d}{dt}{\bar{T}}_{\text{XY}}\left(t\right)=\underset{\text{Δ}t\to 0}{\text{lim}}\frac{E_{\varphi \left|F\left(t+\text{Δ}t\right)\right.}\left[T_{\text{XY}}\left(\varphi \right)\right]-E_{\varphi \left|F\left(t\right)\right.}\left[T_{\text{XY}}\left(\varphi \right)\right]}{\text{Δ}t}\\ =\underset{\text{Δ}t\to 0}{\text{lim}}\frac{E_{\varphi \left|F\left(t\right)G\left(\text{Δ}t\right)\right.}\left[T_{\text{XY}}\left(\varphi \right)\right]-E_{\varphi \left|F\left(t\right)\right.}\left[T_{\text{XY}}\left(\varphi \right)\right]}{\text{Δ}t}. \end{array}$$

Expanding (1) to first order in in Δ*t* and then taking the limit Δ*t* → 0, we arrive, using Mathematica (Wolfram Research, Inc.) for the symbolic algebra, at the following equations for the expected transition counts:

$$\begin{array}{c} \frac{d}{dt}{\bar{T}}_{\text{MM}}\left(t\right)=\mu \frac{bf\left(1-y\right)}{1-gy}-\left(\lambda +\mu \right)a\\ \frac{d}{dt}{\bar{T}}_{\text{MI}}\left(t\right)=-\mu \frac{b\left(1-g\right)}{1-gy}+\lambda \left(1-b\right)\\ \frac{d}{dt}{\bar{T}}_{\text{IM}}\left(t\right)=\lambda a-\mu \frac{f\left(1-g\right)\left(b\left(1-r\right)+cq\right)}{\left(1-gy\right)\left(f\left(1-r\right)+hp\right)}\\ \frac{d}{dt}{\bar{T}}_{\text{DI}}\left(t\right)=\mu \frac{\left(1-g\right)\left(b\left(1-r-hq\right)+cgq\right)}{\left(1-gy\right)\left(f\left(1-r\right)+hp\right)}, \end{array}$$ (5)

with boundary condition

$${\bar{T}}_{\text{XY}}\left(0\right)=\left\{\begin{array}{cc} 1 & \mathrm{X=Y=M}\\ 0 & \text{otherwise} \end{array}\right..$$

The parameters (*a*, *b*, *c*, *f*, *g*, *h*, *p*, *q*, *r*) are defined by (3) for *t* > 0, condition at *t* = 0, where $a\left(0\right)=1$, $f\left(0\right)=1-x$, $g\left(0\right)=x$, $p\left(0\right)=1-y$, $r\left(0\right)=y$, and $b\left(0\right)=c\left(0\right)=h\left(0\right)=q\left(0\right)=0$.

The remaining counts are obtained from (2):

$$\begin{array}{c} {\bar{T}}_{\text{MD}}\left(t\right)=1-{\bar{T}}_{\text{MM}}\left(t\right)-{\bar{T}}_{\text{MI}}\left(t\right)\\ {\bar{T}}_{\text{II}}\left(t\right)={\bar{S}}_{\text{I}}\left(t\right)-{\bar{T}}_{\text{MI}}\left(t\right)-{\bar{T}}_{\text{DI}}\left(t\right)\\ {\bar{T}}_{\text{ID}}\left(t\right)={\bar{T}}_{\text{MI}}\left(t\right)+{\bar{T}}_{\text{DI}}\left(t\right)-{\bar{T}}_{\text{IM}}\left(t\right)\\ {\bar{T}}_{\text{DM}}\left(t\right)=1-{\bar{T}}_{\text{MM}}\left(t\right)-{\bar{T}}_{\text{IM}}\left(t\right)\\ {\bar{T}}_{\text{DD}}\left(t\right)={\bar{S}}_{\text{D}}\left(t\right)+{\bar{T}}_{\text{MM}}\left(t\right)+{\bar{T}}_{\text{IM}}\left(t\right)-{\bar{T}}_{\text{DI}}\left(t\right)-1. \end{array}$$ (6)

The expected state occupancies are governed by the following equations:

$$\frac{d}{dt}{\bar{S}}_{\text{I}}\left(t\right)=\frac{\lambda }{1-x}\left(1+{\bar{S}}_{\text{I}}\left(t\right)\right)$$

$$\frac{d}{dt}{\bar{S}}_{\text{D}}\left(t\right)=\frac{\mu }{1-y}\left(1+{\bar{S}}_{\text{D}}\left(t\right)\right),$$

which, generalizing De Maio (2020), have the closed-form solution

$$\begin{array}{c} {\bar{S}}_{\text{I}}\left(t\right)=\text{exp}\left(\frac{\lambda t}{1-x}\right)-1\\ {\bar{S}}_{\text{D}}\left(t\right)=\text{exp}\left(\frac{\mu t}{1-y}\right)-1. \end{array}$$ (7)

### TKF91 model

When *x* = *y* = 0, our model reduces to the TKF91 model (Thorne *et al.* 1991). Holmes and Bruno (2001) showed that the solution to the TKF91 model can be expressed as a pair HMM of the form shown in Figure 2A, with parameters

$$\begin{array}{l} a\left(t\right)=\left(1-\beta \right)\alpha ,b\left(t\right)=\beta ,c\left(t\right)=\left(1-\beta \right)\left(1-\alpha \right),\\ f\left(t\right)=\left(1-\beta \right)\alpha ,g\left(t\right)=\beta ,h\left(t\right)=\left(1-\beta \right)\left(1-\alpha \right),\\ p\left(t\right)=\left(1-\gamma \right)\alpha ,q\left(t\right)=\gamma ,r\left(t\right)=\left(1-\gamma \right)\left(1-\alpha \right), \end{array}$$

where

$$\begin{array}{c} \alpha \left(t;\lambda ,\mu \right)=\text{exp}\left(-\mu t\right)\\ \beta \left(t;\lambda ,\mu \right)=\frac{\lambda \left(\text{exp}\left(-\lambda t\right)-\text{exp}\left(-\mu t\right)\right)}{\mu \text{exp}\left(-\lambda t\right)-\lambda \text{exp}\left(-\mu t\right)}\\ \gamma \left(t;\lambda ,\mu \right)=1-\frac{\mu \beta }{\lambda \left(1-\alpha \right)}. \end{array}$$

It can readily be verified that this is an exact solution to Equation (3) through Equation (7), when *x* = *y* = 0. Thus, our model reduces exactly to the TKF91 model when indels involve only single residues. In this case, the equivalence $F\left(t+\text{Δ}t\right)=F\left(t\right)G\left(\text{Δ}t\right)$ is exact in the limit Δ*t* → 0.

### Using the model for alignment

To apply this model to sequence alignment, we need to specify a start and end state for G, rather than implicitly assuming infinite-length sequences as we have done up to this point.

A version of G with start and end states is shown in Figure 3A. This can be carried throughout the analysis by also specifying start and end states for F and deriving differential equations for the transitions involving these states. Since this complicates the formulae considerably, we have omitted it. Instead, we propose a heuristic modification of F that includes *ad hoc* transitions from/to start and end states, shown in Figure 3B.

### Parameterizing the model

In principle, the likelihood function is sufficient to parameterize the model: we can compute the gradient numerically to locate the maximum-likelihood parameters, or use Markov Chain Monte Carlo sampling to find the posterior.

In practice, it might be more efficient to use an expectation-maximization algorithm tailored to this model. In the Supplemental Material, we conjecture that a simple expectation-maximization algorithm does exist for this model, and we outline one way it might be arrived at.

### Data availability statement

Our code implementing this model is available under an open-source license at https://github.com/ihh/trajectory-likelihood/tree/benchmark.

File S1 (gzipped tarball) contains JSON files describing the state machines in Figure 2, a Makefile and short script for manipulating these state machines, a Mathematica notebook deriving the equations in this paper, and a text file listing the contents of the tarball. Supplemental material available at figshare: https://doi.org/10.25386/genetics.13040585.

## Results

We implemented the H20 likelihood calculations described in the *Materials and Methods* and those of the models (TKF91, TKF92, MLH04, LG05, RS07, and DM20) reviewed in the *Introduction*. We also implemented a simulator for the underlying indel process, similarly to LAHP19 but using a succinct (run-length encoded) representation of the alignment. We performed simulations at various parameter settings and calculated summary statistics for all methods, including the relative entropy from the simulated to approximate joint distribution *P* (*S_I_*, *S_D_*) and various marginals and moments of this distribution. Our implementations and simulation results are available at https://github.com/ihh/trajectory-likelihood/tree/benchmark.

**Figure 4:**
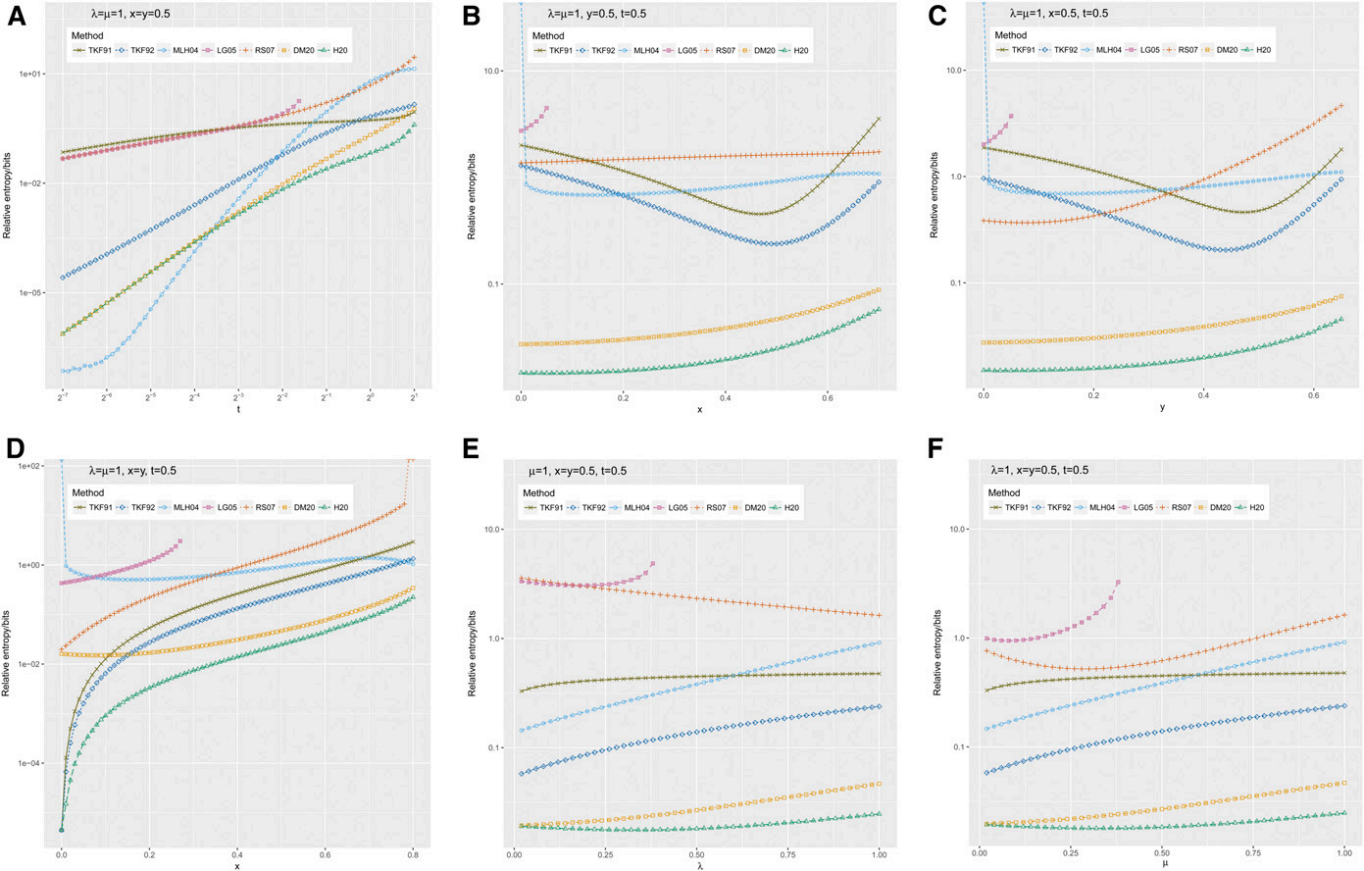
Relative entropies of simulated gap length distributions *P* (*S_I_*, *S_D_*) to the predictions of various approximate methods. The approximation methods are TKF91 (Thorne *et al.* 1991), TKF92 (Thorne *et al.* 1992), MLH04 (Miklós *et al.* 2004), LG05 (Löytynoja and Goldman 2005), RS07 (Redelings and Suchard 2007), and DM20 (De Maio 2020), reviewed in the *Introduction*; and H20 (the present method), defined in the *Materials and Methods*. The simulation procedure is defined in the *Results*. Starting from a parameter setting (*λ* = *μ* = 1, *x* = *y* = 0.5) representative of indel lengths in protein structural alignments, the panels show the following parameter sweeps: (A) varying the time parameter *t* over a range of scales; (B and C) varying the indel extension probabilities *x*, *y* separately, thus exploring irreversible models with insertion-deletion asymmetry; (D) varying *x* and *y* jointly while holding *x* = *y*, exploring reversible models with differing indel lengths; and (E and F) varying the indel rate parameters *λ*,*μ* separately. These are the same experiments (and ordering thereof) shown in Figure 6.

**Figure 6:**
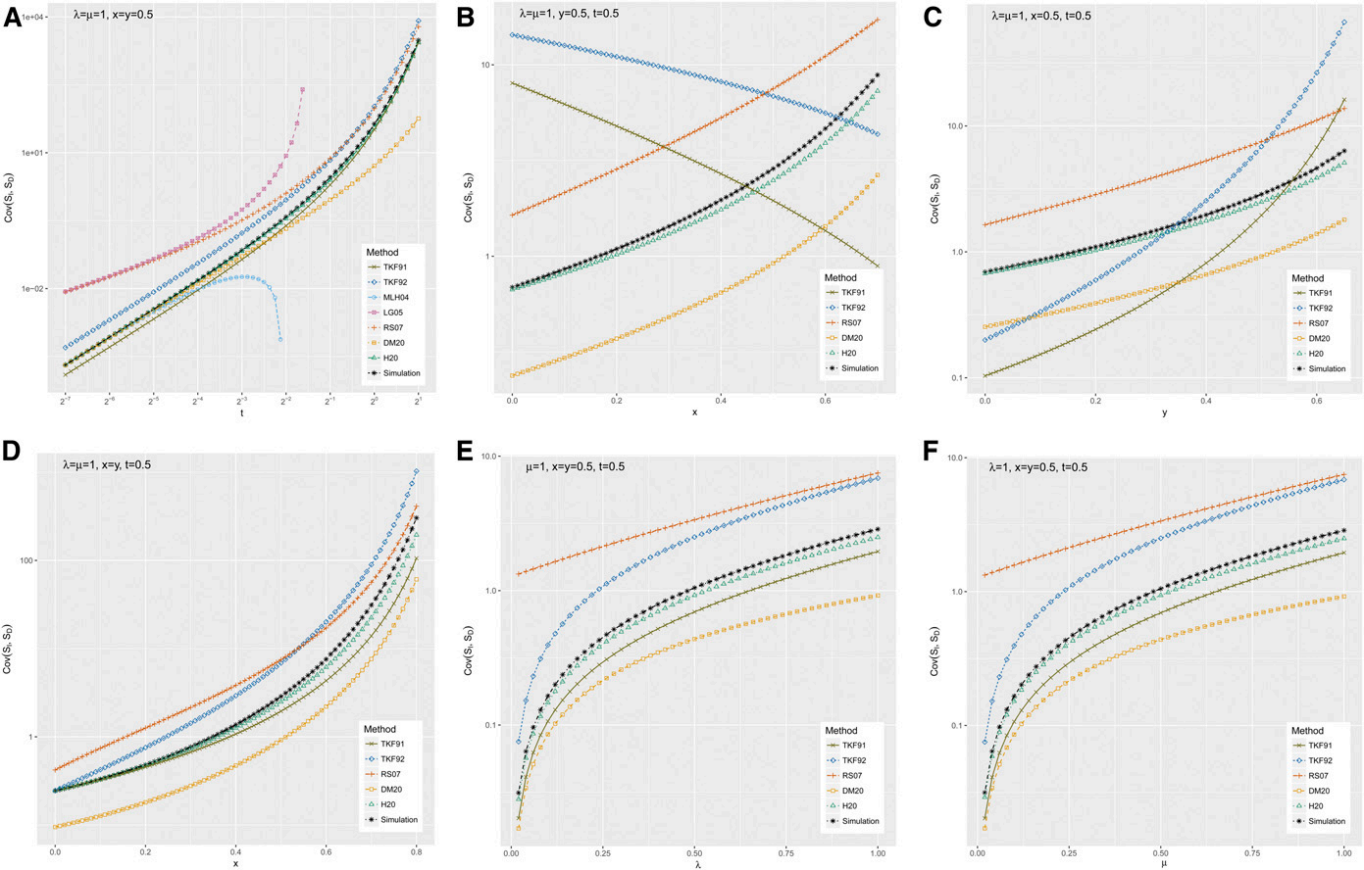
Covariance between the numbers of inserted (*S_I_*) and deleted (*S_D_*) residues in an alignment gap, as revealed by simulation and predicted by various approximate methods. The approximation methods are TKF91 (Thorne *et al.* 1991), TKF92 (Thorne *et al.* 1992), MLH04 (Miklós *et al.* 2004), LG05 (Löytynoja and Goldman 2005), RS07 (Redelings and Suchard 2007), and DM20 (De Maio 2020), reviewed in the *Introduction*; and H20 (the present method), defined in the *Materials and Methods*. The simulation procedure is defined in the *Results*. Starting from a parameter setting (*λ* = *μ* = 1, *x* = *y* = 0.5) representative of indel lengths in protein structural alignments, the panels show the following parameter sweeps: (A) varying the time parameter *t* over a range of scales; (B and C) varying the indel extension probabilities *x*, *y* separately, thus exploring irreversible models with insertion-deletion asymmetry; (D) varying *x* and *y* jointly while holding *x* = *y*, exploring reversible models with differing indel lengths; and (E and F) varying the indel rate parameters *λ*, *μ* separately. These are the same experiments (and ordering thereof) shown in Figure 4.

In Figures 4–6, we show summary statistics for several sweeps of the GGI model parameters (*λ*, *μ*, *x*, *y*, *t*) in the ranges 2^−7^ ≤ *t* ≤ 2^1^, 0 ≤ *x* ≤ 0.7, 0 ≤ *y* ≤ 0.65, 0 ≤ *x* ≤ 0.8 with *y* = x, 0 ≤ *x* ≤ 0.7, 0 < *λ* ≤ 1, and 0 < *μ* ≤ 1. All sweeps are based around the point *λ* = *μ* = 1, *x* = *y* = *t* = 0.5, which was chosen to be indel-symmetric (*λ* = *μ* and *x* = *y*) and representative of amino acid indel lengths: the setting y≃0.5 is consistent with a previous maximum-likelihood estimate based on indel lengths in the HOMSTRAD database (Miklós *et al.* 2004). These parameter sweeps have the effect of exploring each parameter individually, as well as (in the case of the *x*-sweep where *y* = *x*) varying the length of insertions and deletions simultaneously.

To map the GGI model parameters onto those of the models being evaluated, we used the mappings defined by De Maio (2020), with some adjustments to allow for cases where insertions and deletions were asymmetric, which were not reported by De Maio. These mappings are defined in the Supplemental Material. Our criteria for evaluating a model was based on the obviousness of these mappings; so, for example, we did not include models significantly more parameter-rich than GGI (Rivas and Eddy 2015), where to define a mapping would have required so many choices as to have created a new model.

In each experiment we performed *N* simulations on a sequence of length *L* and estimated gap sizes up to *G* residues, discounting gaps at the end of the sequence (which have different statistics). In most cases these settings were *L* = 10^3^ and *G* = 10^2^, with *N* = 10^7^ for *t* < 2^−5^, *N* = 10^6^ for 2^−5^ ≤ *t* < 2^−4^, and *N* = 10^5^ for 2^−4^ ≤ *t* < 1. For *t* ≥ 1, and also for *y* > 0.6, we set *L* = 10^5^, *G* = 10^3^, and *N* = 10^2^. The higher *N* at low *t* was to ensure sufficient sampling of infrequent events over short evolutionary timespans. The higher values of (*G*, *L*) at high (*t*, *y*) were to mitigate end effects as deletions become longer (which happens as *t* or *y* get large). Because of the $O\left(L\right)$ time complexity of finding a position in a sequence and then inserting or deleting elements, the simulation time is $O\left(NL^{2}t\right)$ yielding $O\left(NLt\right)$ indel events. Thus, when increasing *L*, we also reduced *N*. The time required for simulation handily dominated the total CPU time taken by the experiment, in almost all cases; the longest-running data points of Figure 4A each required over 10 min on our late-2014 iMac (4GHz quad-core Intel i7 CPU, 32GB 1600MHz DDR3 RAM). Even with these run times, the observed counts were zero for a majority of the (*S*_I_,*S*_D_) tuples in many cases. This illustrates a fundamental problem with the purely simulation-based LAHP19 approach; running it enough times to sample all cases is impractical. This may be one reason why the authors of LAHP19 limited the maximum gap length *G* to 50 residues (Levy Karin *et al.* 2019). A potential solution to this problem would be to use a limited sample to fit a parametric model, although we have not explored that approach. Of course, other indel simulation programs may run faster than ours.

**Figure 7:**
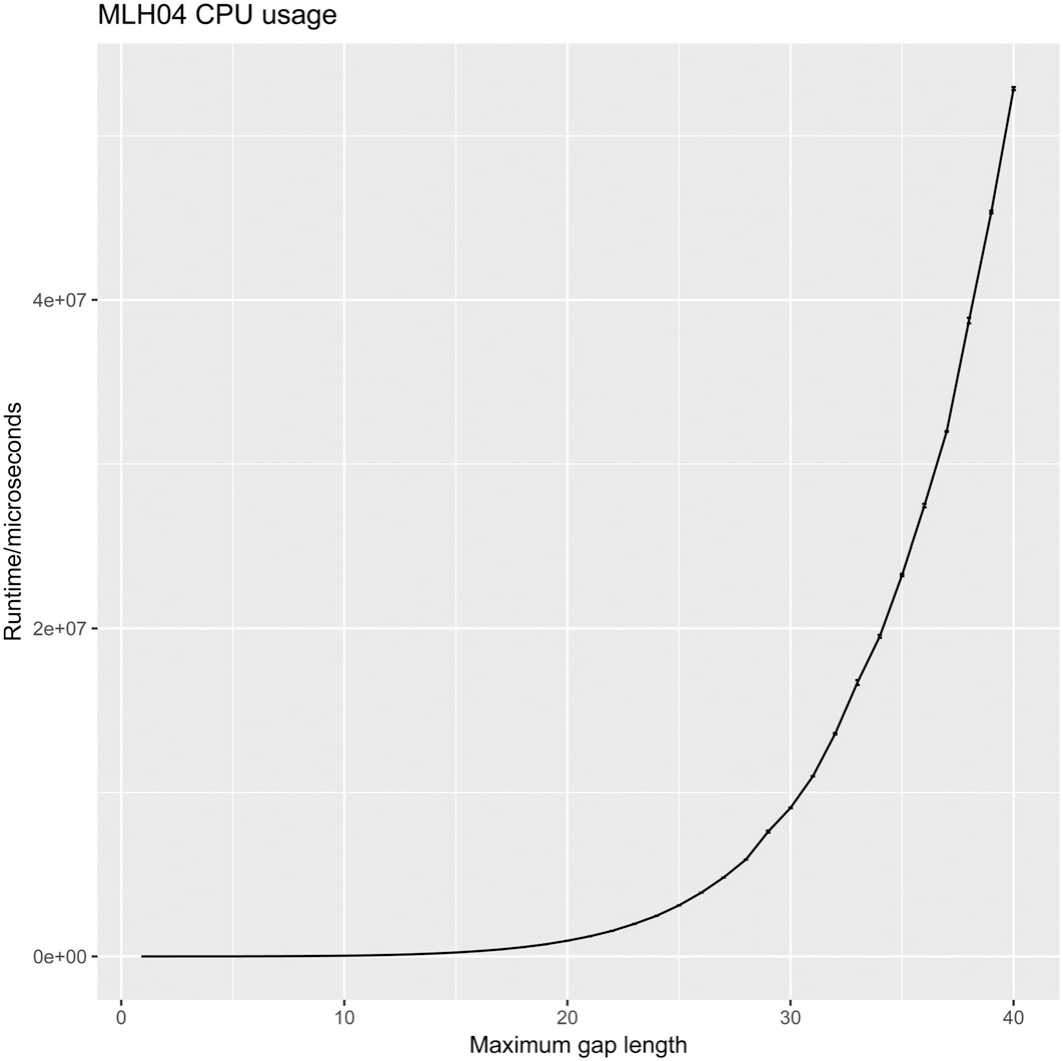
The running time of the MLH04 method is prohibitive and increases steeply for long gaps, since it requires the explicit enumeration of all intermediate indel states in the trajectory (Miklós *et al.* 2004). In this plot, each data point is an average of 100–1000 repetitions on a late-2014 iMac (4GHz quad-core Intel i7 CPU, 32GB 1600MHz DDR3 RAM). The running times of DM20 and H20 are negligible in comparison (< 1 ms).

For the MLH04 method, we limited *G* to 30 in all cases, since it takes impractically long to calculate likelihoods of longer gaps. This is illustrated by Figure 7, which plots the runtime of MLH04 as a function of gap length. To calculate likelihoods of gap lengths up to 30 residues at a particular parameter setting, MLH04 takes roughly 10 sec on current desktop hardware, which is vastly slower than the microsecond-scale runtime of all other methods (with the exception of direct simulation, which requires many repetitions to achieve statistical accuracy). Because we only considered shorter gaps for MLH04, when calculating the relative entropy from the simulated gap length distribution, we used the truncated distribution $P\left(S_{I},S_{D}\left|S_{I}\leq G,S_{D}\leq G\right.\right)$ to avoid infinities that would otherwise occur due to MLH04 assigning zero probability to longer gaps.

Figure 4 shows the relative entropy (Kullback–Leibler divergence) between the simulated and various approximated distributions for the six parameter sweeps: *t*, *x*, *y* (separately and together), *λ*, and *μ*. Starting with the time sweep in Figure 4A, we see a pattern that was broadly repeated in time sweeps across other parameterizations (data not shown): at very short times, the MLH04 trajectory-enumerating approximation is the best fit to the simulated distribution (with the proviso that it can only handle short gaps, and takes significant time to compute). At these low times, the DM20 and H20 approximations are almost indistinguishable, and are the second-best fit; TKF92 is the next best after that, followed by the PRANK and BAliPhy HMMs. However, when *t* gets large enough (in Figure 4A it occurs around t≳0.4), the divergence of MLH04 shoots up, to the point where it quickly becomes the worst or second-worst fit. This is presumably because, at higher *t*, there is a significant probability of having more than three events in the trajectory (MLH04 is limited to three events due to the combinatorial complexity of enumerating longer trajectories). This leaves DM20 and H20 as the best methods. Shortly after this point, DM20 and H20 start to separate, so that H20 has a slight edge over DM20. Meanwhile, LG05 (the PRANK HMM) is not defined for all parameterizations, since it contains probabilities proportional to 1 − 2*δ* where $\delta =1-\exp\left(-\frac{\lambda t}{1-x}\right)$. Thus, at high enough *t* or *x*, we have *δ* > 0.5 and so the probabilities become negative. The RS07 HMM (used by BAliPhy) remains defined but becomes inaccurate at high *t*. It should be noted that the time values *μt* > 1 − *y* correspond to a scenario where the sequence is saturated with deletions, which may be of less relevance to many applications in alignment and phylogenetics. However, it is at the borderline of this region where the differences between the methods are most pronounced.

Seeking to explore these differences further, we varied *x* and *y* in Figure 4, B and C. The general trend is consistent: H20 and DM20 are the most accurate methods, LG05 is not defined for most of this parameter regime, and the other methods cluster in a pack, affected to varying degrees by the parameter sweep. TKF92 (which models multiresidue indels) is consistently a better fit than TKF91 (which does not), as might be expected. Both TKF91 and TKF92 explicitly assume reversibility between insertions and deletions, and appear to be quite strongly affected by deviations from symmetry.

When *x* and *y* are varied together, as in Figure 4D, we see quite different behavior across the different approximation methods. In the special case *x* = *y* = 0, when indel events can include only a single residue, the GGI model is essentially identical to TKF91. Unsurprisingly TKF91, TKF92, and H20—which all admit exact solutions in this special case—have effectively zero relative entropy. By contrast, the MLH04 model performs very poorly when *x* = *y* = 0, since this parameterization requires at least *K* separate events to explain a gap of length *K*, and so MLH04 assigns zero probability to any gap of length ≥3, yielding an infinite Kullback–Leibler divergence from the simulated distribution. As soon as *x* and *y* become nonzero, the MLH04 divergence becomes finite again, as (from the other direction) do those of TKF91, TKF92, and H20. All relative entropies continue to rise as the mean indel length increases, MLH04 rising most slowly. Eventually, H20’s error approaches DM20’s error from below. At x≳0.78, the RS07 probabilities—which drop off rapidly with increasing gap length—are rounded to zero by floating-point precision errors, including for some gap lengths that are reached by the simulation, leading to infinities in the relative entropy for RS07.

Varying *λ* and *μ* (Figures 4, E and F) reveals that H20, DM20, TKF92, and MLH04 rise monotonically in inaccuracy as these rate parameters increase from zero. TKF91 is a worse approximation than all these methods when *λ* = 0 or *μ* = 0, but stays mostly flat as they are increased, to the point where it eventually beats MLH04 as an approximation. RS07’s inaccuracy decreases monotonically with *λ*, but has a minimum as a function of *μ*. LG05, as with the other sweeps, performs weakly and is only defined for part of the parameter regime. One notable point is that H20 and DM20 have almost identical accuracy when *λ* = 0 or *μ* = 0, but diverge as those rates increase, with H20 performing better than DM20.

To summarize Figure 4: at all parameterizations, H20 is more accurate than DM20, and is the most accurate of all the three-state pair HMMs. H20 is outperformed only by MLH04, and then only at very low values of *t* and for very short gaps (taking a very long time to compute). In the parameter range where most alignment occurs (μt≪1), DM20 and H20 are roughly equivalent; they diverge as gaps become very frequent. Of the closed-form approximations, TKF92 is most accurate, but is significantly less reliable than H20.

**Figure 5:**
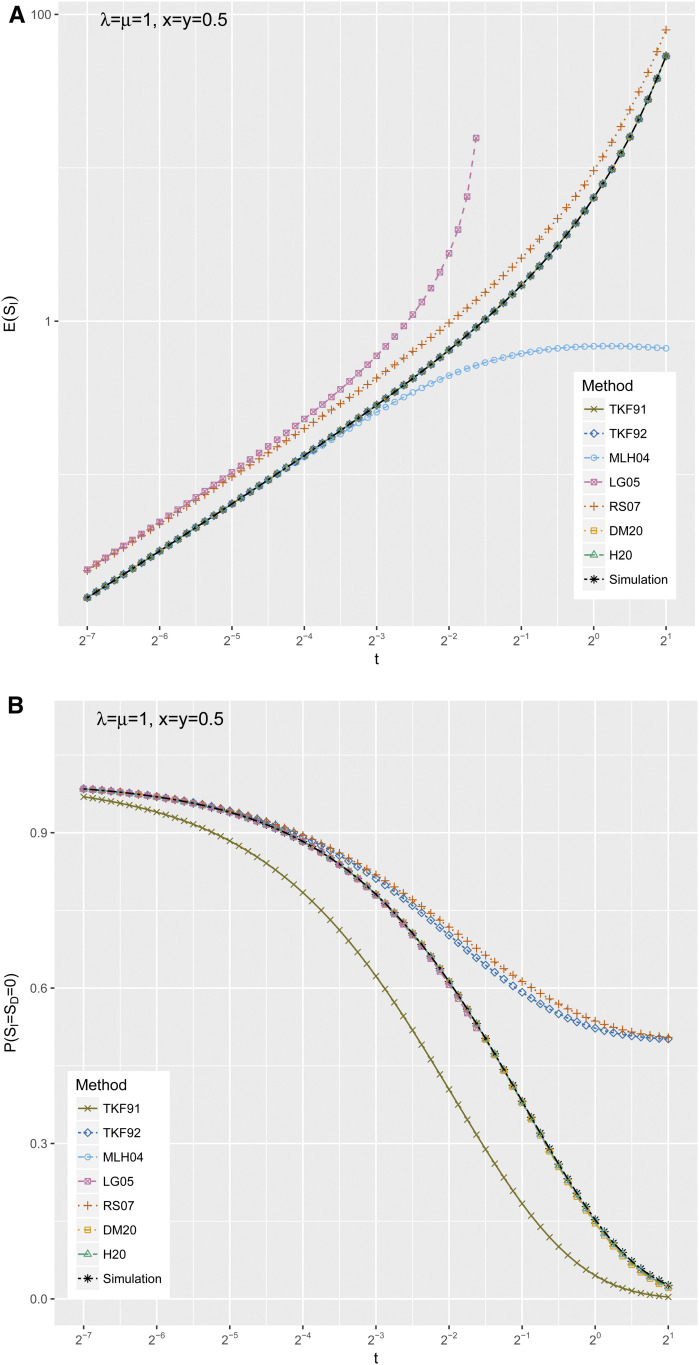
Moments of the gap length distribution *P* (*S_I_*,*S_D_*) as revealed by simulation, compared to the predictions of various approximate methods. The approximation methods are TKF91 (Thorne *et al.* 1991), TKF92 (Thorne *et al.* 1992), MLH04 (Miklós *et al.* 2004), LG05 (Löytynoja and Goldman 2005), RS07 (Redelings and Suchard 2007), and DM20 (De Maio 2020), reviewed in the *Introduction*; and H20 (the present method), defined in the *Materials and Methods*. The simulation procedure is defined in the *Results*. The parameter range explored is *λ* = *μ* = 1, *x* = *y* = 0.5, and 2^−7^ ≤ *t* ≤ 2^1^, corresponding to panel A of Figures 4 and 6. Panel A shows the expected insertion length as a function of time, and panel B shows the probability that there is no gap between adjacent ancestral residues.

To understand these differences better, we examined moments of the simulated and approximate distributions. These are plotted in Figures 5 and 6.

Figure 5A plots the expected insertion length, focusing on the *t*-parameter sweep (similar trends were apparent in the other sweeps). All the methods that find explicit closed-form formulae for the expected insertion length as a continuous-time process (which is to say TKF91, TKF92, DM20, and H20) show an exact fit to the simulated distribution, while RS07 deviates more significantly, and LG05 as previously noted is only defined for part of the time range. MLH04 underestimates the expected insertion length significantly after $t≳2^{-3}$, again presumably because MLH04 is a poor approximation when there are multiple expected indel events per observed alignment gap.

Figure 5B plots the probability that adjacent ancestral residues have no gap between them, *P*(*S_I_* = *S_D_* = 0). As with Figure 5A, this is plotted as a function of time; and once again, DM20 and H20 closely match the simulation, while RS07 is an overestimate. However, for this statistic (unlike the expected insertion length), MLH04 and (where defined) LG05 are as accurate as DM20 and H20, while TKF91 underestimates the probability and TKF92 overestimates it.

To summarize the results plotted in Figure 5, the expected insertion length and empty-gap probability are uninformative as to the differences between DM20 and H20: both methods seem to get these statistics right. However, noting that the main difference between the DM20 and H20 is that DM20 does not allow transitions back and forth between the I and D states, instead requiring deletions to precede insertions, we might expect the covariance between insertion and deletion lengths to be a better diagnostic.

Figure 6 plots the insertion-deletion covariance for all parameter sweeps, using the same ordering of subfigures (one per parameter sweep) as the relative entropy plots in Figure 4. What we see in these plots is that the relative accuracy of the covariances for DM20 and H20 closely tracks the relative entropies of their gap length distributions. In the time sweep, DM20 and H20 perform identically at low times, but gradually diverge; in the *x* and *y* sweeps, they are separated throughout the parameter range; and in the *λ* and *μ* sweeps, they are close when the rate parameter is zero, but then diverge rapidly. The most significant single factor determining the covariance between insertion length *S_I_* and deletion length *S_D_* is the joint probability *P*(*S_I_* = *S_D_* = 0) that both are zero; however, Figure 5B shows that both DM20 and H20 basically get this probability correct. The most obvious remaining factor that might explain the different covariances is the absence of an *I* → *D* transition in the DM20 pair HMM, which De Maio explicitly suggested might be a potential limitation on the accuracy of DM20 (De Maio 2020). Thus, we propose this as an explanation for the improved performance of H20, while noting that this improvement is relatively small.

As for the covariance of the other methods, the results in Figure 6 are broadly consistent with Figure 4, although they do not provide as a coherent single explanation of the differences between methods, as is the case when comparing DM20 and H20. Two points are worth remarking on. First, in Figure 6A, the covariance of MLH04 dips sharply around $t≳2^{-3}$, and in fact eventually becomes negative (although it is not shown on this plot, due to the logarithmic *y*-axis). The negative covariance can be explained by MLH04’s restriction to a small finite number of indel events in any given trajectory, which implies that every insertion event is one event that cannot be a deletion, and vice versa. The other point worth noting is that, as is the case with the relative entropies, Figure 6D clearly shows that TKF91, TKF92, and H20 are an exact fit to the simulated data when *x* = *y* = 0, which reduces the GGI model to the TKF91 model.

We can summarize all the simulation results as follows. In virtually all cases, H20 is the most accurate of pair HMM methods, outperformed only by MLH04 when the rate of indels is slow enough that there is negligible probability that an observed alignment gap can be explained by multiple overlapping indel events. DM20 is very close behind H20; the differences between DM20 and H20 appear to be explainable in terms of H20’s better modeling of covariation between insertion and deletion lengths, which is probably attributable to an additional *I* → *D* transition in the H20 pair HMM.

## Discussion

We have shown that an evolutionary model which can be represented infinitesimally as an HMM can be formally connected to a pair HMM that approximates its finite-time solution. This may be viewed as an automata-theoretic framing of the Chapman–Kolmogorov equation.

Ours is a coarse-graining technique. It is generally the case that composing two state machines will yield a more complex machine, since the composite state space is the Cartesian product of the components. We approximate this more complex machine with a renormalizing operation that eliminates null states and maps the remaining states of each type back to a single representative state in the approximator.

We used this approach to derive ODEs for the transition probabilities of a minimal (three-state) pair HMM that approximates a continuous-time indel process with geometrically distributed indel lengths. We have implemented numerical solutions to these equations, and demonstrated that they outperform the previous best methods. The improvement in accuracy over DM20, the closest method, is small but significant; further, our approach puts the process of deriving the approximation on a more systematic footing. In the Supplemental Material, we also conjecture similar ODEs for the posterior expectations of the sufficient statistics that would be required to fit this model by expectation maximization, although we have not yet tested this approach.

Point substitution models are the foundation of likelihood phylogenetics. There is, additionally, a substantial literature combining such models with HMMs and stochastic context-free grammars, for the purposes of genome annotation and other sequence analysis. Indels are a potential annotation signal: phase-preserving indels, for example, are a common signature of protein-coding selection. As well as being useful tools to represent and (approximately) solve such models, automata theory can be used to build sampling kernels (Redelings and Suchard 2007) and reconstruct ancestral sequences (Westesson *et al.* 2012).

Our emphasis on the GGI model, a continuous-time Markov process defined on sequences of residues, somewhat disadvantages models like TKF92, which technically defines a process on sequences of multiresidue fragments. We have argued that there is no evidence such indivisible fragments really exist, so instead we evaluated TKF92 as an approximation to the GGI model. However, the routine usage of amino acid fragment models to predict protein tertiary structure suggests a valid counterargument: such models may usefully capture some aspects of selection. Further, TKF92 can be generalized in other ways, allowing for richer models of fragment mutation; for example, to model the evolution of RNA structure. In this context, it is promising that our method recovers TKF91 (and therefore TKF92) as special cases.

Input-output automata are well suited to modeling indels in statistical phylogenetics. It seems possible that the method of this paper might be applied to other instantaneous rate models of local evolution where the infinitesimal generator can be represented as an HMM. It is tempting to speculate that a similar approach may also be productively applied to stochastic context-free grammars, so as to analyze RNA; or to model literary texts, phonemes, vocabularies, music, source code, bird song, or other alignable sequences that evolve by local edits over time.
